# Supplementary material for: Inflammation and Tumor Progression: The Differential Impact of SAA in Breast Cancer Models
Source: Biology (Basel). 2024 Aug 23;13(9):654. doi: 10.3390/biology13090654 (PMC11429026; doi:10.3390/biology13090654)
Supplement: Supplementary file 1 [file biology-13-00654-s001.zip › Supplementary File S1.pdf]

Supplementary File S1

CPGR | CENTRE FOR PROTEOMIC & GENOMIC RESEARCH

	Analytical Report 2
Report to	Dr Tanja Davis<br>Department of Physiological Sciences<br>Mike de Vries Building<br>c/o Merriman and Bosman Street<br>Stellenbosch<br>7600
Document Number	03092020-001_PCR
Version	1
Status	Final
Date	3 September 2020
Project ID	1373PCR_A_DAVIS_GENO
Author	Aubrey Shoko<br>RT-PCR Platform Manager
Reviewer	Moleboheng Seutloali<br>Senior Genomics Technician
Authorized By	Aubrey Shoko<br>RT-PCR Platform Manager

Table of Contents

1. Background and samples submitted 3

2. Analysis 3

2.1. DNA yield and quality 3

2.2. Genotyping of mouse tails 6

3. Experimental procedures 6

3.1. PCR primers 6

3.2. DNA extractions 7

3.3. Genotyping 7

4. Deviations 7

5. Enquiries 7

1. Background and samples submitted

Fourty-four mouse tail tissue samples (listed in Table S1) were submitted for genotyping of transgenic mice. The samples were submitted along with nucleotide sequences of the genotyping primers which were to be ordered by the CPGR.

2. Analysis

2.1. DNA yield and quality

The yield and purity of the extracted DNA samples were assessed using the NanoDrop-8000 spectrophotometer (Table S1) and the integrity of the samples was assessed using agarose electrophoresis (Figure S1).

Table S1. DNA concentrations, OD ratios ($A_{260/280}$ and for DNA extractions from the $A_{260/230}$) mouse tail tissues.

#	Sample	Conc.	Units	260/280	260/230
1	MT01	84.14	ng/ul	1.84	2.02
2	MT02	91.41	ng/ul	1.9	2.16
3	MT03	48.52	ng/ul	1.82	2.05
4	MT04	41.68	ng/ul	1.82	2.03
5	MT05	41.09	ng/ul	1.82	1.74
6	MT06	96.48	ng/ul	1.9	2.13
7	MT07	63.96	ng/ul	1.9	2.12
8	MT08	65.77	ng/ul	1.92	1.98
9	MT09	67.17	ng/ul	1.91	1.79
10	MT10	126.9	ng/ul	1.89	1.97
11	MT11	32.43	ng/ul	1.84	2.47
12	MT12	30.49	ng/ul	1.96	2
13	MT13	66.57	ng/ul	1.91	2.01

#	Sample	Conc.	Units	260/280	260/230
14	MT14	172.2	ng/ul	1.91	2.17
15	MT15	55.57	ng/ul	1.92	1.64
16	MT16	49.28	ng/ul	1.88	2.04
17	MT17	33.65	ng/ul	1.91	2.16
18	MT18	99.41	ng/ul	1.91	2.08
19	MT19	67.89	ng/ul	1.87	2.36
20	MT20	75.15	ng/ul	1.94	2.12
21	MT21	103.2	ng/ul	1.96	2.22
22	MT22	95.51	ng/ul	1.89	2.17
23	MT23	64.42	ng/ul	1.92	2.25
24	MT24	91.69	ng/ul	1.89	2.06
25	MT25	252.3	ng/ul	1.95	2.22
26	MT26	133.8	ng/ul	1.95	2.21
27	MT27	123.5	ng/ul	1.94	2.03
28	MT28	123.4	ng/ul	2	1.81
29	MT29	113.3	ng/ul	1.97	2.32
30	MT30	80.33	ng/ul	1.94	2.2
31	MT31	77.91	ng/ul	2.02	2.22
32	MT32	122.2	ng/ul	1.95	2.21
33	MT33	89.23	ng/ul	1.93	2.14
34	MT34	96.29	ng/ul	1.94	2.22
35	MT35	78.81	ng/ul	1.96	2.12

#	Sample	Conc.	Units	260/280	260/230
36	MT36	99.96	ng/ul	1.97	2.08
37	MT37	119.2	ng/ul	1.95	2.21
38	MT38	125	ng/ul	1.93	2.16
39	MT39	88.48	ng/ul	1.97	1.87
40	MT40	115.5	ng/ul	1.95	2.15
41	MT41	75.59	ng/ul	1.96	2.03
42	MT42	118.6	ng/ul	1.9	2.07
43	MT43	92.99	ng/ul	2.01	1.9
44	MT44	98.44	ng/ul	1.97	2.22

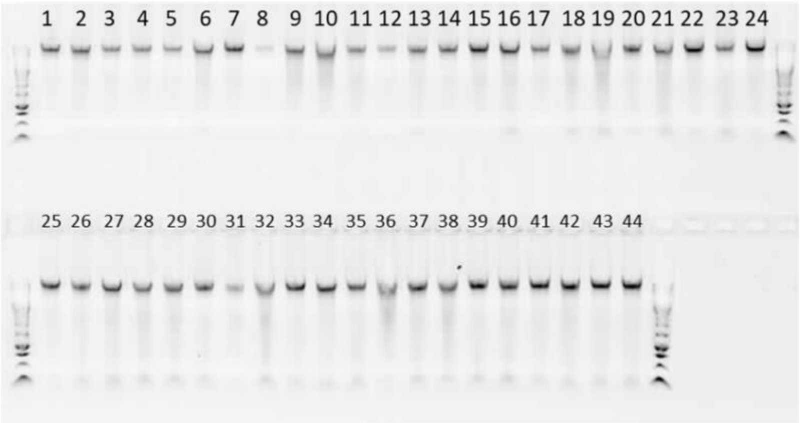

Figure S1. 1% Agarose gel electrophoresis images of high molecular weight genomic DNA ex-tracted from mouse tails.

2.2. Genotyping of mouse tails

The 303 bp fragment was amplified in all 44 DNA samples tested (Figure S2).

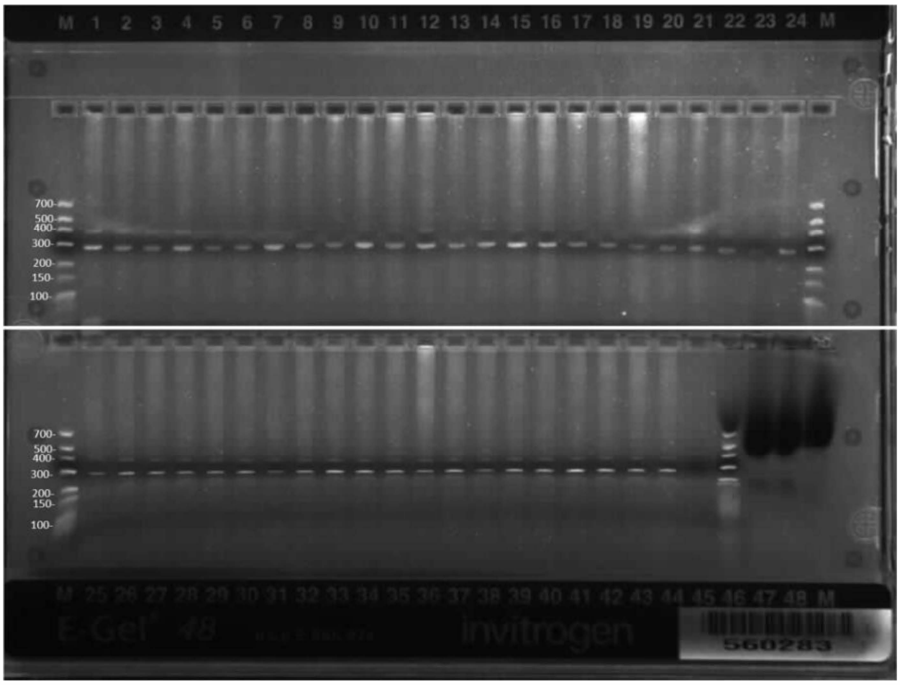

Figure S2. Genotype identification by PCR amplification of mouse tail DNA samples. Amplifica-tion of the ~303 bp target fragment was amplified in PCR reactions using 150ng of each DNA sample (lanes 1-44). An NTC (no-template control) was included (lane 45) as a negative con-trol. The Thermo Scientific Gene Ruler Low Range DNA Ladder was used as a size standard.

3. Experimental procedures

3.1. PCR primers

The three PCR primers were sourced from Inqaba Biotech and were resuspended in TE buffer (10mM Tris, pH 8.0, 1mM EDTA) buffer to a 100µM stock concentration, as per manufacturer instructions. Working stocks of 10µM were prepared and used for the PCR reactions.

3.2. DNA extractions

Genomic DNA extraction from the mouse tails was automated using the QIAamp® DNA Kit (QIAGEN) on the QIAcube and eluted in 100 µl. The quality and quantity of the extracted DNA was measured using the NanoDrop-8000 spectrophotometer. Ratio of the absorbance at A_{230}, A_{260} and A_{280} were estimated to determine the quality and the DNA concentration was measured based on A_{260} values. Ten µL of each DNA sample was analysed on a 1% agarose gel stained with ethidium bromide.

3.3. Genotyping

For PCR, the three primers were mixed (0.3 µM for each primer) with 150 ng each DNA sample and amplified with the KAPA HiFi PCR Kit in a 25 µL total reaction volume. The cycling protocol was: 1 cycle of 95°C for 3 min; 35 cycles of 98°C for 20 sec, 60°C for 15 sec, and 72°C for 30 sec. PCR products were separated by electrophoresis using E-Gel® 48 4% gels.

4. Deviations

No deviations to protocols or standard methods employed or deviations from the agreed study plan.

5. Enquiries

Your application specialist on this project is Aubrey Shoko; do not hesitate to contact him on 021 447 5669 or aubrey.shoko@cpgr.org.za for additional discussion or information on this report.
