# Supplementary material for: Inflammation and Tumor Progression: The Differential Impact of SAA in Breast Cancer Models
Source: Biology (Basel). 2024 Aug 23;13(9):654. doi: 10.3390/biology13090654 (PMC11429026; doi:10.3390/biology13090654)
Supplement: Supplementary file 1 [file biology-13-00654-s001.zip › Supplementary File S2.pdf]

Supplementary File S2

RNA EXTRACTION, CDNA SYNTHESIS AND CYCLE SEQUENCING

Report date: 2022-03-18 *Reported by: AA Vorster*

CAF Central Analytical Facilities
STELLENBOSCH UNIVERSITY

BACKGROUND

The biological specimen, EO771, was received for RNA extraction and mRNA enrichment on the 8th of February 2022.

MRNA ENRICHMENT

mRNA was isolated from 2.5 x 10^4 cells using the Dynabeads®mRNA DIRECT™Micro Kit (ThermoFisher, Waltham, MA, USA) according to the manufacturer's protocol for "*mRNA isolation from cultured cells and cell suspensions*, *p10*" (Revision 004, Revision Date: 14 May 2012). Briefly, cells were washed in 250ul phosphate-buffered saline and resuspended in 100ul Lysis/Binding Buffer. The lysate was mixed with Dynabeads® Oligo (dT)25 to allow mRNA with poly(dA) tails to anneal to the beads. Unbound RNA was washed from the supernatant using wash buffer A and B. The bound mRNA was subsequently eluted in 100ul ice-cold 10mM Tris-HCl. The procedure was performed in duplicate.

CDNA SYNTHESIS

The SuperScript™ VILO™ cDNA Synthesis Kit (ThermoFisher Scientific) was used to convert expressed RNA transcripts into a representative cDNA library according to the manufacturer's protocol (Doc. Part No. 100002284 Pub. No. MAN0000749 Rev. A.0). Briefly, 10ul enriched mRNA (8.5ng/ul) was transcribed with the addition of 4ul 5X VILO™Reaction Mix and 2ul 10X SuperScript™ Enzyme Mix in a final reaction volume of 20ul. cDNA was synthesized at 42°C for 1 hr, followed by inactivation at 85°C at 5 minutes.

QUALITY CONTROL

The total RNA samples were assessed for RNA integrity (RINe) on the TapeStation 4150, using the RNA ScreenTape assay (Agilent Technologies, Waldbronn, Germany) according to the protocol, G2991-90021 Rev. B. The quality and quantity of mRNA yielded from the isolation method was considered sufficient for first-strand cDNA synthesis. The concentration of the cDNA was determined with fluorometry on the Qubit 4.0 using the Qubit™ssDNA Assay Kit (ThermoFisher Scientific).

Table 1. RNA quality control assessment

Sample	RINe	RNA conc.	cDNA conc.
EO771_Replicate1	8.9	8.5 ng/ul	72.4 ng/ul
EO771_Replicate2	8.7	11 ng/ul	65.0 ng/ul

PCR AMPLIFICATION

For PCR amplification of single-plex SAA targets, 50ng cDNA was added to a final reaction volume of 20ul; consisting of 10 pmol of each sense and antisense primer pair, 250uM of each dNTP, 1x Phusion HF buffer and one unit Phusion™ High-Fidelity DNA Polymerase. Amplification of the cDNA template was performed using a touch-down PCR (Green & Sambrook, 2018), where the target template was dissociated at 98°C for the first 10 amplification cycles, followed by 25 cycles of amplification with a lower DNA dissociation temperature of 89°C. Primer annealing temperatures for SAA1, SAA2 and SAA3 was 55 °C, whilst the SAA4 primers were annealed at 60°C. The yield and specificity of the amplified products was verified on the PerkinElmer LabChip® GXII Touch (PerkinElmer, Waltham, MA, USA), using the X-mark chip and HT DNA NGS 3K reagent kit according to the manufacturer's protocol; CLS145098 Rev. E.

POST-PCR PURIFICATION

Post-PCR purification was performed with a 1.8x volume Agencourt™AMPure™XP (Beckman Coulter, Brea, CA, USA) reagent and eluted in 20ul nuclease-free H_2O.

SEQUENCING

Direct-cycle sequencing was performed according to standard protocols.

REFERENCES

Green MR, Sambrook J. Touchdown Polymerase Chain Reaction (PCR). Cold Spring Harb Protoc. 2018 May 1;2018(5). doi: 10.1101/pdb. prot095133. PMID: 29717053.

Figure S3. Materials and methods of EO771 SAA1/2/3/4 mRNA expression analysis by the Stellenbosch University Central Analytical Facilities (CAF).

Table S2. Primers sourced from Integrated DNA Technologies (IDT) and used for PCR amplification and direct-cycle Sanger sequencing.

	Forward primer (5' – 3')	Reverse primer (5' – 3')
**SAA1**	CAT TTG TTC ACG AGG CTT TCC	GTT TTT CCA GTT AGC TTC CTT CAT GT
**SAA2**	TCT TCT GCT CCC TGC TCCT	AGC CAG CTT CCT TCA TGT CA
**SAA3**	ACA GCC AAA GAT GGG TCC AG	CTG GCA TCG CTG ATG ACT TT
**SAA4**	TGTCCTCTGTTCTTTGTTCCTG	TGGTCTGCATTTTGGTAATTAGC

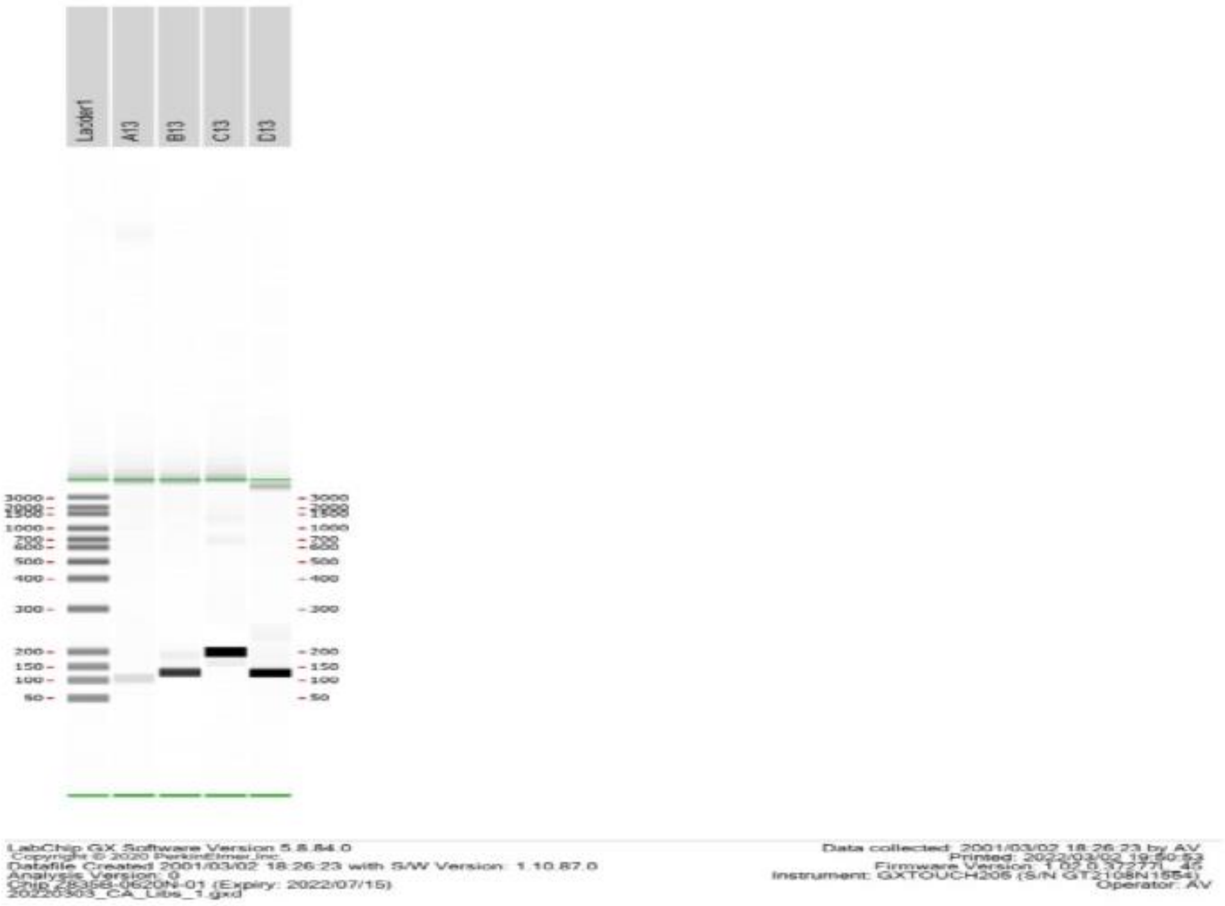

Figure S4. Virtual electrophoretic gel of SAA1/2/3/4 PCR amplicons. Lane A13, SAA1 (~101 bp); Lane B13, SAA2(~129 bp); Lane C13, SAA3 (~198 bp); Lane D13, SAA4 (~125 bp).

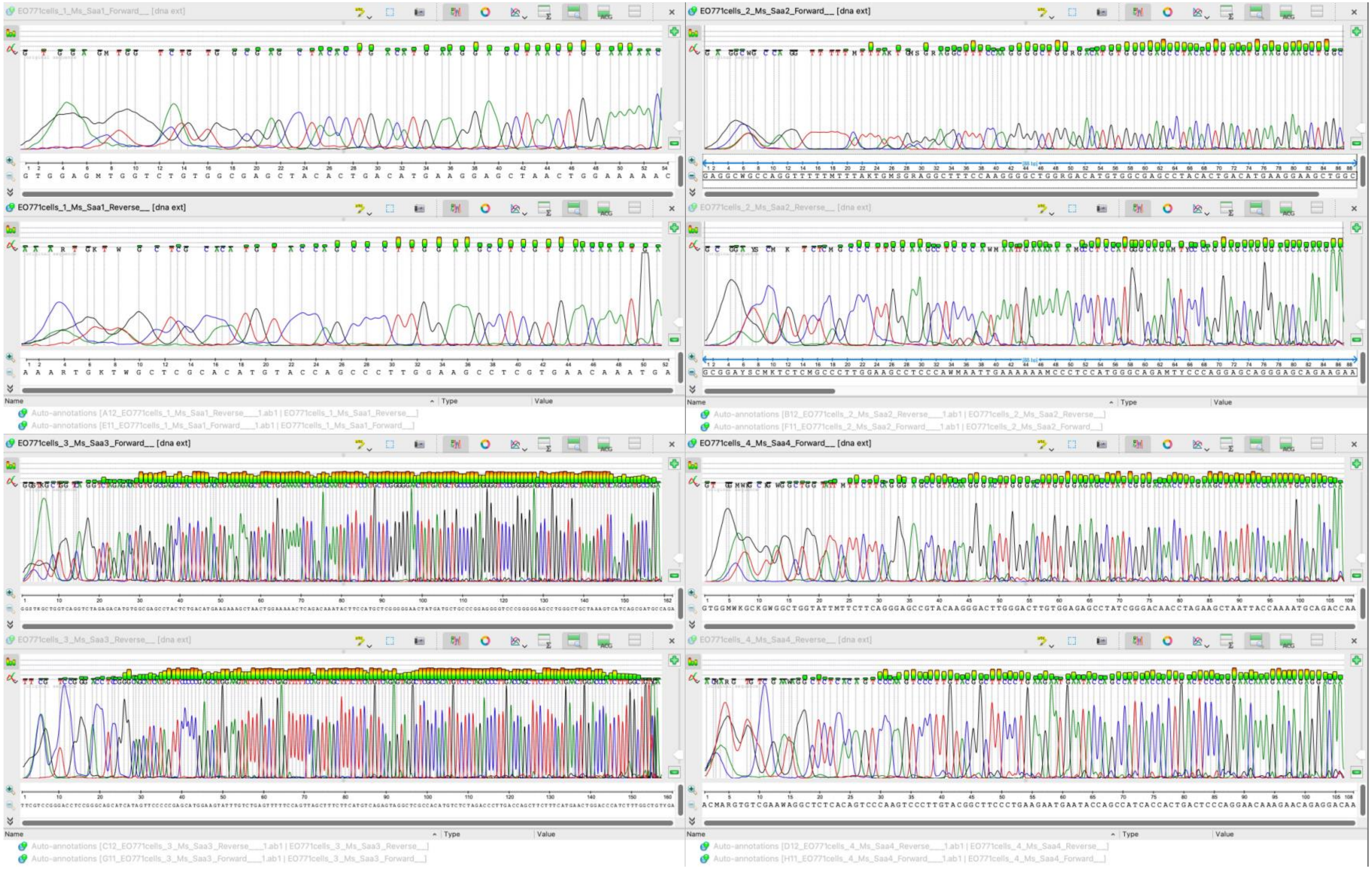

Figure S5. Electropherograms of PCR amplicon direct-cycle Sanger sequencing.

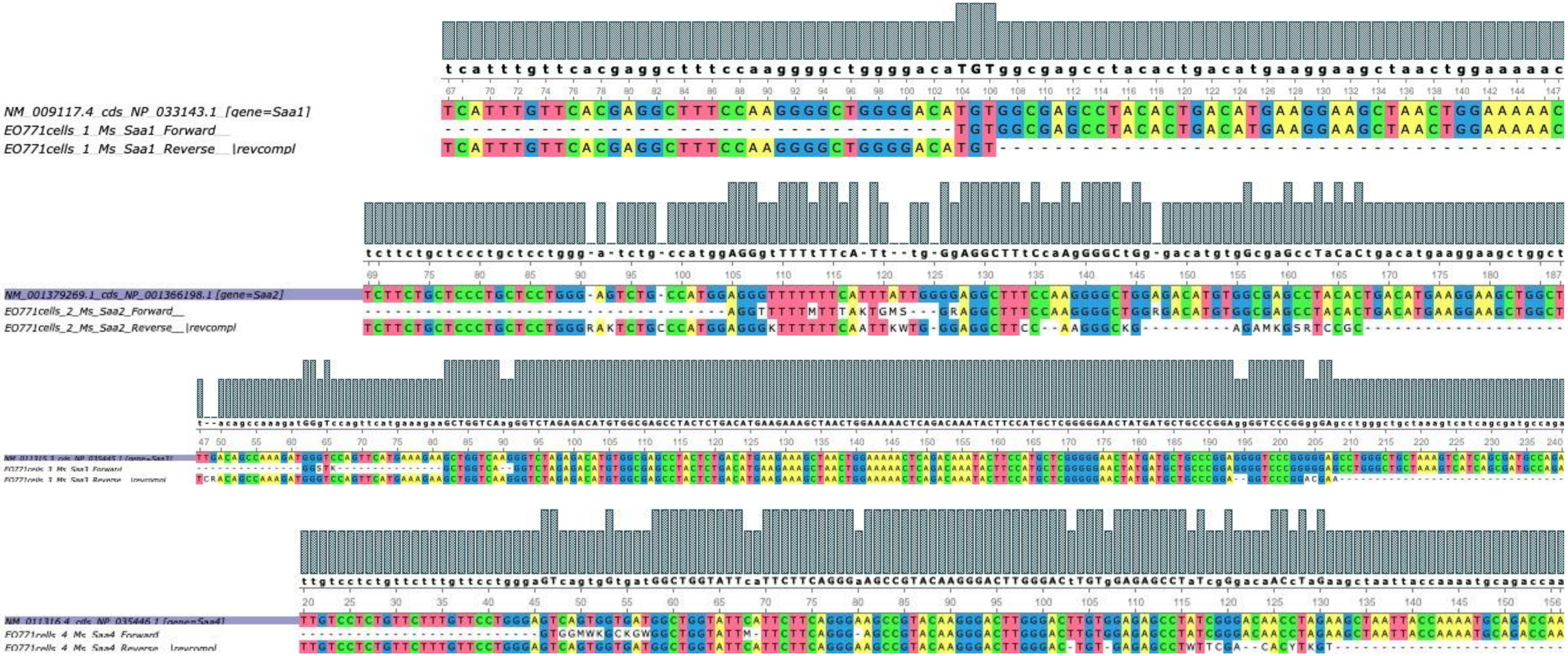

Figure S6. MUSCLE (Unipro UGENE) forward and reverse sequence alignments of direct-cycle Sanger sequence results to NM_009117.4 (SAA1), NM_001379269.1 (SAA2), NM_011315.3 (SAA3), and NM_011316.4 (SAA4) coding sequences. From top to bottom: SAA1, SAA2, SAA3, and SAA4.

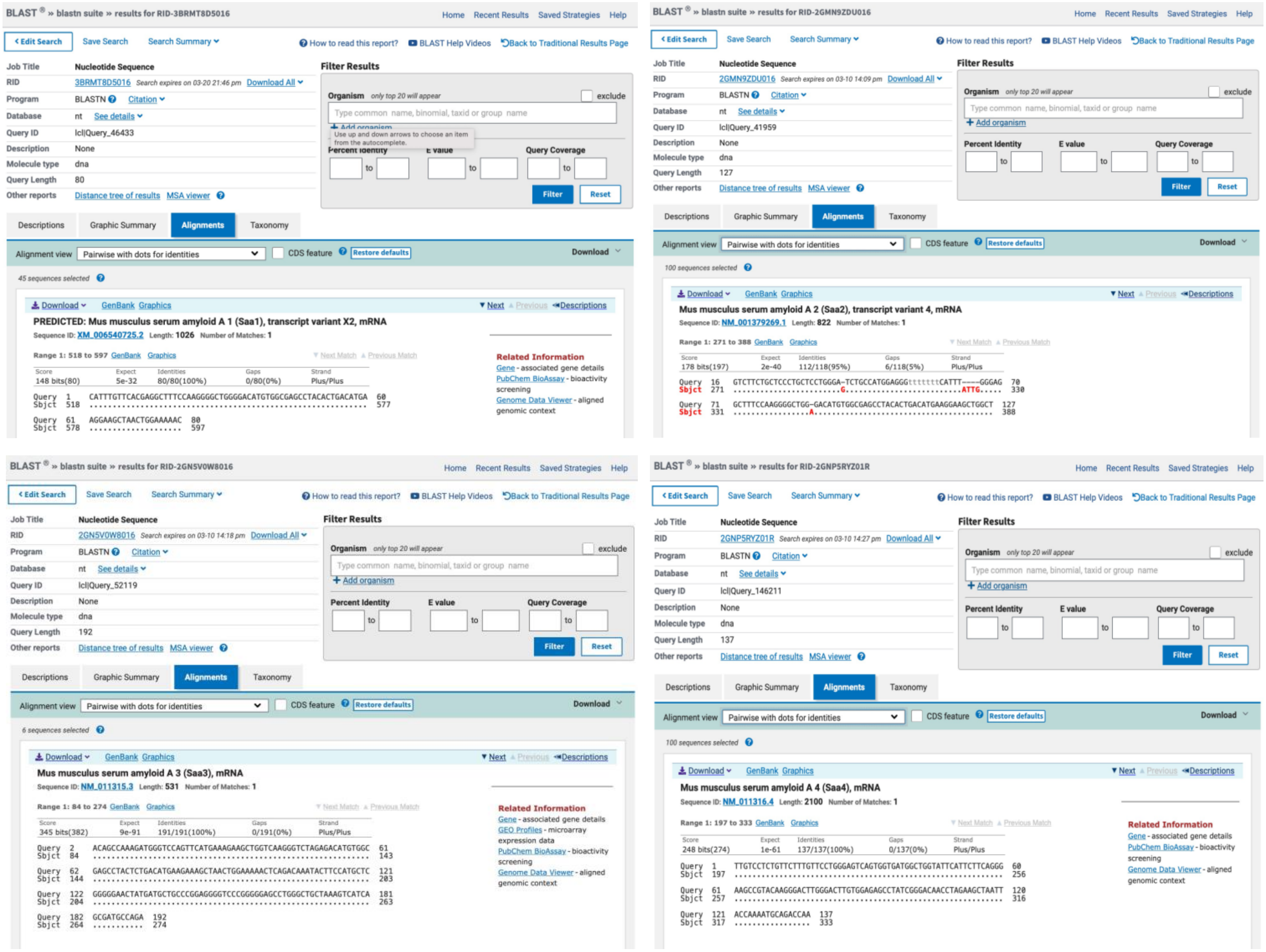

Figure S7. BLAST results of SAA1/2/3/4 consensus sequences determined from Figure S7.
