# Supplementary material for: Inflammation and Tumor Progression: The Differential Impact of SAA in Breast Cancer Models
Source: Biology (Basel). 2024 Aug 23;13(9):654. doi: 10.3390/biology13090654 (PMC11429026; doi:10.3390/biology13090654)
Supplement: Supplementary file 1 [file biology-13-00654-s001.zip › Supplementary FIle S3.pdf]

Supplementary File S3

Table S3. Antibodies used in Western blotting

Primary antibody	MW (kDa)	Dilution	Time	Secondary antibody	Dilution	Time (min)
**SAA antisera**	~ 12	1:1000	1 Day	Anti-mouse HRP	1:10 000	60
**β-catenin**	~ 92	1:1000	1 Day	Anti-rabbit HRP	1:1000	60
**Laminin 1β**	~ 210	1:200	1 Day	Anti-rat HRP	1:5000	60
**Vimentin**	~ 57	1:1000	1 Day	Anti-rabbit HRP	1:10 000	60
**E-cadherin**	~ 135	1:1000	1 Day	Anti-mouse HRP	1:10 000	60
**Snail**	~ 29	1:1000	1 Day	Anti-rabbit HRP	1:10 000	60
**αSMA**	~ 42	1:1000	1 Day	Anti-mouse HRP	1:10 000	60
**tCAS3 & cCAS3**	~ 19, 30	1:1000	3 Days	Anti-rabbit HRP	1:10 000	60
**tCAS7 & cCAS7**	~ 20, 35	1:1000	1 Day	Anti-rabbit HRP	1:10 000	60
**tCAS8 & cCAS8**	~ 18, 57	1:1000	1 Day	Anti-rabbit HRP	1:10 000	60
**tCAS9 & cCAS9**	~ 35, 47	1: 500	1 Day	Anti-mouse HRP	1:10 000	60
**Cytochrome C**	~ 14	1:1000	1 Day	Anti-rabbit HRP	1:10 000	60
**tPARP & cPARP**	~ 89, 116	1:1000	1 Day	Anti-rabbit HRP	1:10 000	60
**MCM2**	~ 125	1:1000	1 Day	Anti-rabbit HRP	1:10 000	60
**p53**	~ 53	1:2500	1 Day	Anti-mouse HRP	1:10 000	60
**p16**	~ 16	1:2500	1 Day	Anti-rabbit HRP	1:10 000	60
**NLRP3**	~ 110	1:1000	1 Day	Anti-rabbit HRP	1:10 000	60
**NFκB & p-NFκB**	~ 65	1:1000	1 Day	Anti-rabbit HRP	1:10 000	60

SAA, serum amyloid a; α-SMA, α-smooth muscle actin; tCAS, total (full length) caspase; cCAS, cleaved caspase; tPARP, total (full length) poly (ADP-ribose) polymerase (PARP); cPARP, cleaved PARP; MCM2, DNA mini-chromosome maintenance protein 2 or DNA replication licensing factor 2; p53, tumor protein 53; p16, cyclin-dependent kinase inhibitor 2A; NLRP3, nucleotide-binding oligomerization domain-like receptor with pyrin domain 3; NFκB, nuclear factor kappa-light-chain-enhancer of activated B cells; p-NFκB, phosphorylated NFκB.

De Beer laboratory: Mouse SAA antisera; Abcam: Laminin 1β (ab44941), tCAS8/cCAS8 (ab25901), MCM2 (ab108935), p53 (ab26), p16 (ab189034), α-SMA (ab7817), Anti-rat HRP (ab205720); Cell Signalling Technology: β-actin (4967), β-catenin (19807), Vimentin (5741), E-cadherin (14472), Snail (3879), tCAS3/cCAS3 (9661, 9662), tCAS7/cCAS7 (12827), tCAS9/cCAS9 (9508), Cytochrome C (11940), tPARP/cPARP (9532), NLRP3 (15101), NFκB (8242), p-NFκB (3033), Anti-rabbit HRP (7074), Anti-mouse HRP (7076).
