# Supplementary material for: Inflammation and Tumor Progression: The Differential Impact of SAA in Breast Cancer Models
Source: Biology (Basel). 2024 Aug 23;13(9):654. doi: 10.3390/biology13090654 (PMC11429026; doi:10.3390/biology13090654)
Supplement: Supplementary file 1 [file biology-13-00654-s001.zip › Supplementary FIle S4.pdf]

Supplementary File S4

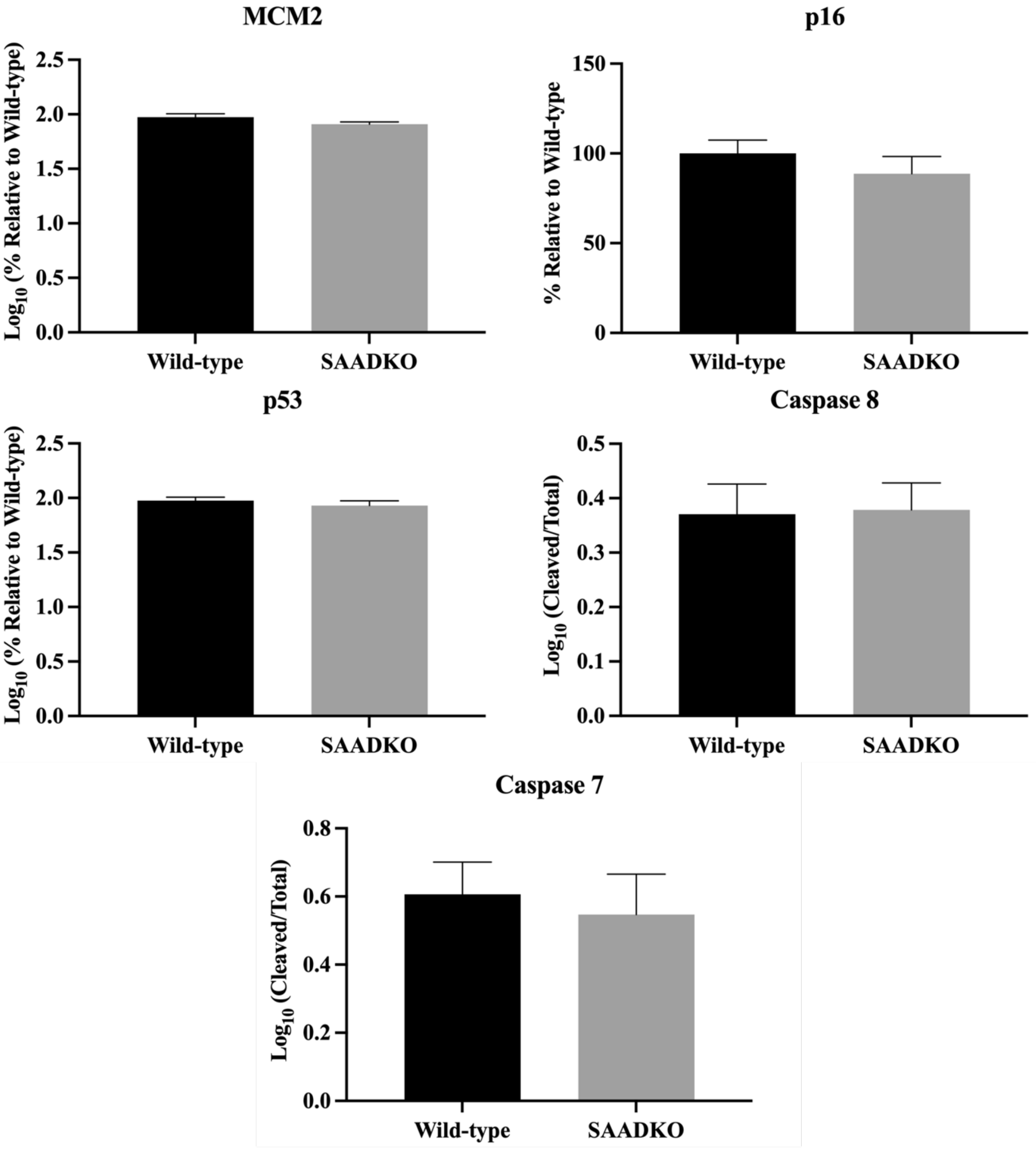

Figure S8. Tumor apoptotic (caspase 7, caspase 8) and cell cycle markers (p53, p16, MCM2) that were not influenced by systemic SAA1/2-deficiency. Data represent mean ± SEM (N = 7). Caspase 7, caspase 8, p53, and p16 were analyzed with an uncorrected unpaired t-test. MCM2 was analyzed with an unpaired t-test with Welch’s correction. Representative western blot images can be found in Figure S12.

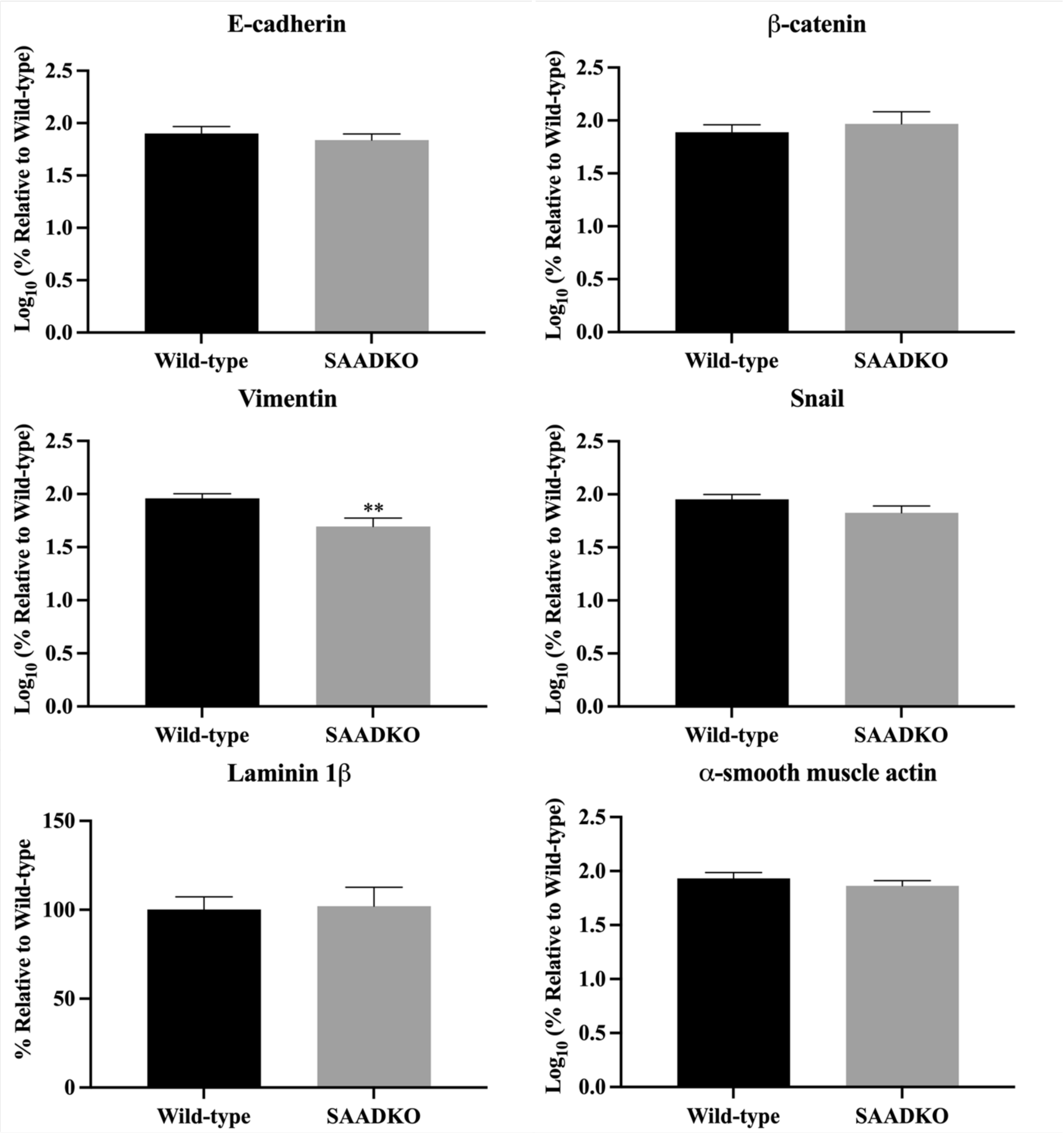

Figure S9. SAADKO tumors only express lower levels of the EMT marker, vimentin. Data represent mean ± SEM (N = 7). E-cadherin, Laminin 1β, and α-smooth muscle actin were analyzed with an uncorrected unpaired t-test. Vimentin and β-catenin were analyzed with an unpaired t-test with Welch's correction. Snail was analyzed with a Mann-Witney test. ** $p < 0.01$ WT vs. SAADKO. Representative western blot images can be found in Figure S12.

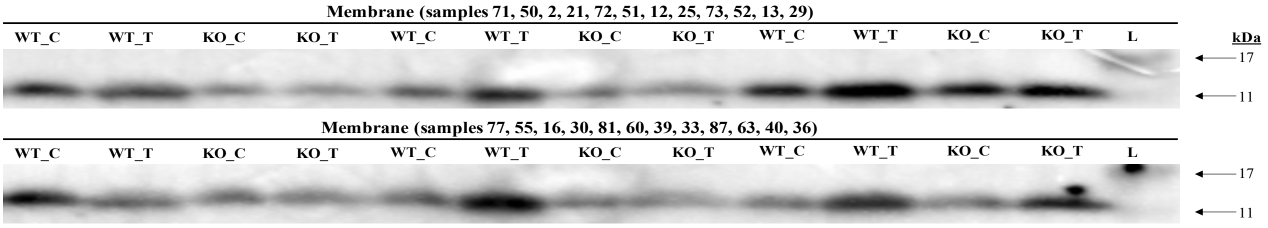

Figure S10. Representative western blot images for plasma SAA from WT control, WT tumor, SAADKO control and SAADKO tumor mice. Sample numbers indicated above each membrane correspond to the order of loading. Representative total protein (stain-free) images are shown in Fig. D.4. WT_C, Wild-type control; WT_T, Wild-type tumor; KO_C, SAA1/2 double-knockout control; KO_T, SAA1/2 double-knockout tumor; L, molecular weight marker.

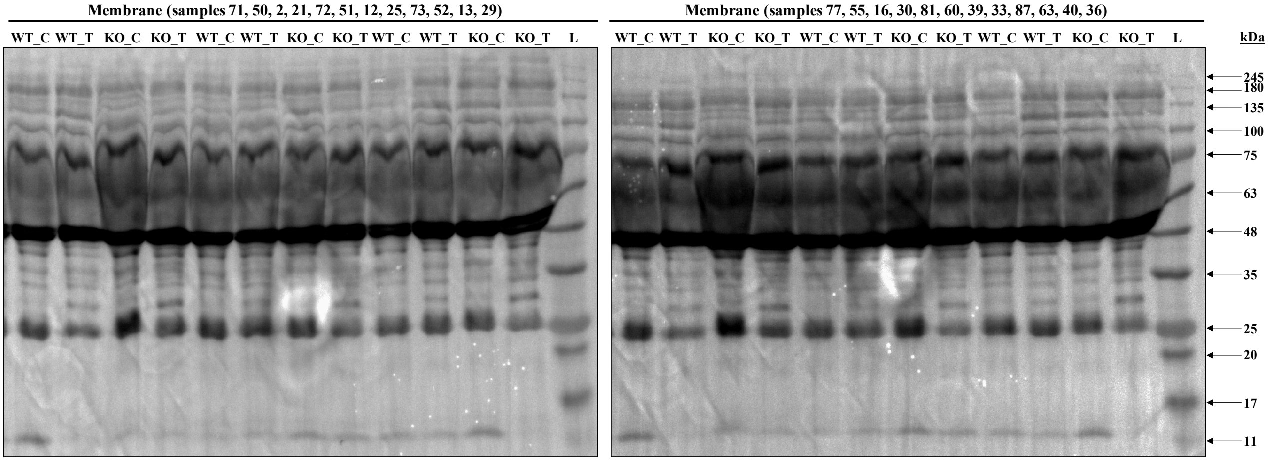

Figure S11. Representative total protein (stain-free) images for plasma of WT control, WT tumor, SAADKO control and SAADKO tumor mice. Sample numbers indicated above each membrane correspond to the order of loading. WT_C, Wild-type control; WT_T, Wild-type tumor; KO_C, SAA1/2 double-knockout control; KO_T, SAA1/2 double-knockout tumor; L, molecular weight marker.

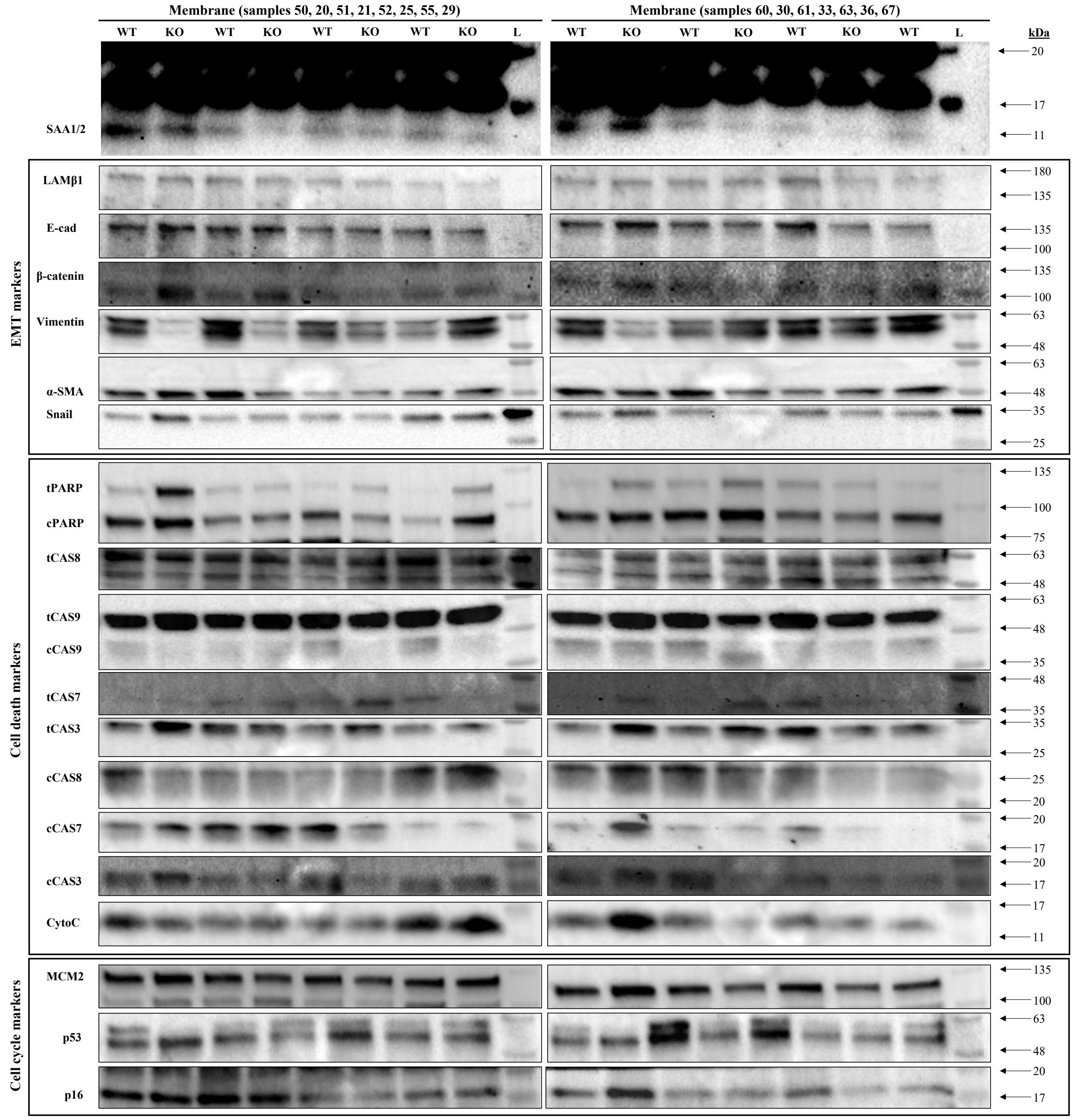

Figure S12. Representative western blot images of proteins blotted for WT and SAADKO tumor lysates. Sample numbers indicated above each membrane correspond to the order of loading. Representative total protein (stain-free) images are shown in Figure S13. WT, Wild-type; KO, SAA1/2 double-knockout; LAMβ1, laminin 1β; E-cad, E-cadherin; α-SMA, α-smooth muscle actin; cCAS, cleaved caspase; tCAS, total caspase; cPARP, cleaved PARP; tPARP, total PARP; CytoC, cytochrome C; L, molecular weight marker.

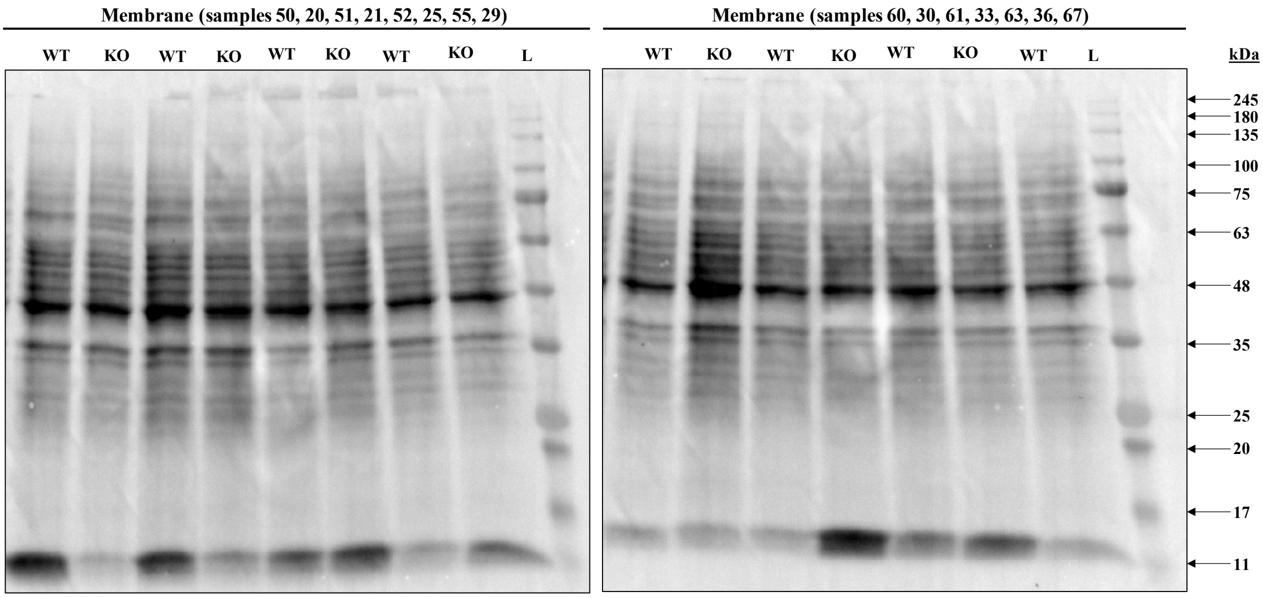

Figure S13. Representative total protein (stain-free) images for tumor lysates of WT and SAADKO mice. Sample numbers indicated above each membrane correspond to the order of loading. WT, Wild-type; KO, SAA1/2 double-knockout; L, molecular weight marker.

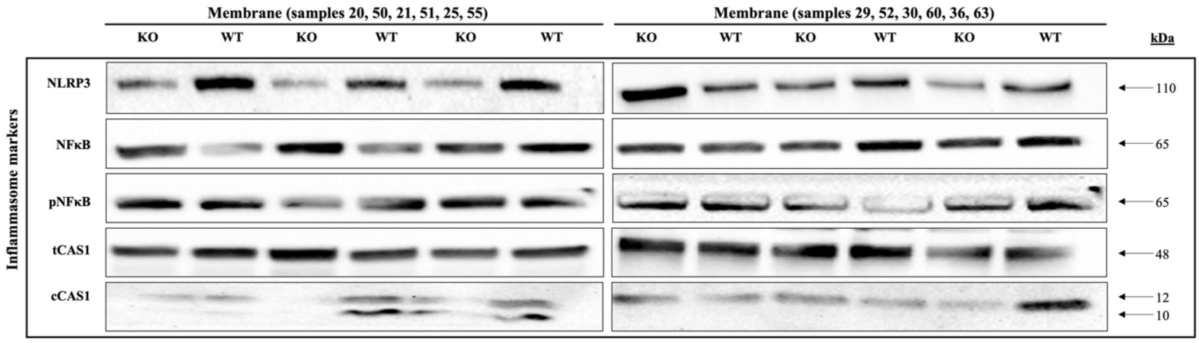

Figure S14. Representative western blot images of proteins blotted for WT and SAADKO tumor lysates. Sample numbers indicated above each membrane correspond to the order of loading. Representative total protein (stain-free) images are shown in Figure S15. WT, Wild-type; KO, SAA1/2 double-knockout; NLRP3, nucleotide-binding oligomerization domain-like receptor with pyrin domain 3; NFκB, nuclear factor kappa-light-chain-enhancer of activated B cells; p-NFκB, phosphorylated NFκB; cCAS, cleaved caspase; tCAS, total caspase.

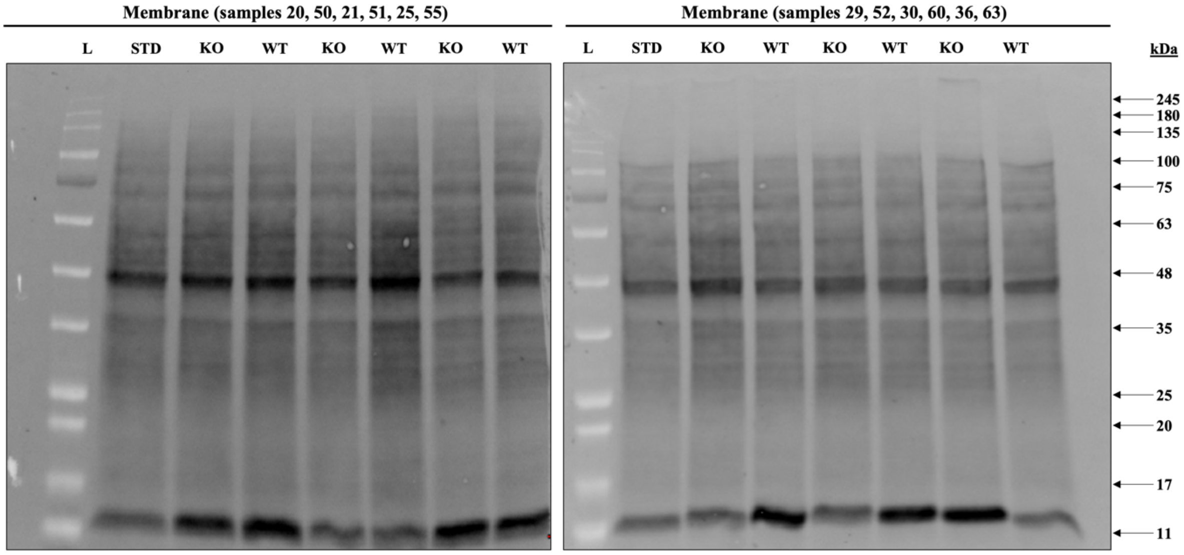

Figure S15. Representative total protein (stain-free) images for tumor lysates of WT and SAADKO mice. Sample numbers indicated above each membrane correspond to the order of loading. WT, Wild-type; KO, SAA1/2 double-knockout; L, molecular weight marker; STD, standard.
